# Recent Advances in Food Waste Transformations into Essential Bioplastic Materials

**DOI:** 10.3390/molecules29163838

**Published:** 2024-08-13

**Authors:** Abdulmoseen Segun Giwa, Ehtisham Shafique, Nasir Ali, Mohammadtaghi Vakili

**Affiliations:** 1 School of Civil and Environmental Engineering, Nanchang Institute of Science and Technology, Nanchang 330108, China; giwaabdulmoseensegun@ncpu.edu.cn; 2 Department of Biological and Health Sciences, Pak-Austria Fachhochschule: Institute of Applied Sciences and Technology, Khanpur Road Haripur 22621, Pakistan; ehtishamshafique94@gmail.com; 3 Orlen Unicre a.s., Revolǔcní 1521/84, 400 01 Ústí nad Labem, Czech Republic

**Keywords:** kitchen waste, bioplastics production, consolidated bioprocessing, lignocellulose depolymerization, polyhydroxyalkanoates (PHA), renewable aromatics

## Abstract

Lignocellulose is a major biopolymer in plant biomass with a complex structure and composition. It consists of a significant amount of high molecular aromatic compounds, particularly vanillin, syringeal, ferulic acid, and muconic acid, that could be converted into intracellular metabolites such as polyhydroxyalkanoates (PHA) and hydroxybutyrate (PHB), a key component of bioplastic production. Several pre-treatment methods were utilized to release monosaccharides, which are the precursors of the relevant pathway. The consolidated bioprocessing of lignocellulose-capable microbes for biomass depolymerization was discussed in this study. Carbon can be stored in a variety of forms, including PHAs, PHBs, wax esters, and triacylglycerides. From a biotechnology standpoint, these compounds are quite adaptable due to their precursors’ utilization of hydrogen energy. This study lays the groundwork for the idea of lignocellulose valorization into value-added products through several significant dominant pathways.

## 1. Introduction

A significant amount of daily kitchen waste (KW) was produced due to expanding urbanization, population growth, and rates of food utilization. Food waste is defined as “food losses of quality and quantity occurring in the supply chain at the production, post-harvest, and processing stages, including industrial and agri-food wastes”, by the Food and Agriculture Group. Household kitchen waste, however, contributes to the worldwide waste generated by food that typically begins during the early four predictable stages: “purchase, storage, cooking, and serving, or in other words, food that was bought but never cooked, served, or consumed, or the food waste generated during cooking, or all four stages”. Improper storage facilities frequently serve as catalysts for the production of food waste statistics; each year, about 1.3 billion tons of food waste are thrown away globally [1] These wastes include leftover food, such as waste from food preparation, vegetables, fruits, bread, and dairy products coming from a variety of sources, including homes and restaurants [2].

In particular, in Asian countries, rapid urban and economic growth is expected to lead to an increase in the amount of kitchen garbage. China, the economic powerhouse of Asia, produces almost 1.9 billion dollars and 105 tons of organic trash annually. Additionally, more than 35 million tons of food (6% of the world’s total food to feed 30 to 50 million people) is produced annually from China [3]. Many countries, including Korea, Australia, Mexico, the US, and India, have followed suit, and now the annual food waste generated by these countries is around 624 to 3500 tons. Approximately USD 218 billion worth of food is wasted each year [4,5]. In the United Kingdom alone, about 15 million tons of food waste are generated each year. Food waste and kitchen waste significantly deplete other resources like land, labor, power, water, etc. [6].

The development of kitchen trash is a concern for the majority of countries, including Canada. The reason is that cooking waste contains hazardous organic compounds in high concentrations that come along during the production, storage, shipping, and distribution stages, which produce foul odors and unhealthy gases [7]. Moisture in the kitchen waste causes leachate upon dumping, which requires additional wastewater treatment systems to treat the leachate. Kitchen trash can also be employed as fertilizer and food for animals. However, the salt content of local cuisine hampers the utilization of leftover food as animal nutrition and fertilizer, affecting both the economy and the climate vastly [4]. To effectively utilize kitchen garbage to produce sustainable sources of energy requires new applications and opportunities to be investigated so that significant financial and energy losses can be prevented [1].

It is reported that the French government suggests and promotes the utilization of kitchen waste to produce energy (in the form of biogas) and value-added products like bioplastics, etc. [8]. It was projected that this idea would save USD 167 billion while reducing 88 million tons of food waste annually if adopted. Similarly, natural products like proteins, amino acids, and organic acids present in household waste can be used as feedstock and crucial minerals through enzymatic transformations or microbial fermentation. Several publications documented the use of biofuel production methods, operational factors, and novel bacteria in fermentation, even if several of them documented the contemporaneous conversion of KW to energy [9]. The direct use of domestic kitchen food waste for the possible synthesis of polyhydroxyalkanoates (PHA) has received very little attention from researchers.

The main reason synthetic polymers are used so frequently is that they can withstand both physical and chemical alterations. However, their failure to disintegrate upon completion of their intended function causes disposal issues and is a significant factor in the global problem of plastic pollution. The use of biopolymers, on the other hand, has been proposed as a viable solution to this problem [10]. However, they should fulfill requirements like quick degradation both during and after usage, along with high quality and durability. Then, PHAs are instances of polyesters with the highest degree of degradability when compared to various other biopolymers.

In 2018, the production of biopolymers was just 2.01 million tons, or 0.5% of the total amount of plastic produced worldwide, which is anticipated to increase by 2.4 million tons by 2023 [10]. Lastly, increasing the market share of bioplastics can help to lessen reliance on fossil fuels, and advancements in the circular economy can facilitate the shift to a bio-based society. With this perspective, the current review fills the knowledge gap and provides insight to clarify PHA synthesis procedures based on food processing wastes. This article’s first section focuses on the composition of KW and recent research that uses all or part of KW to produce PHA. There is also a synopsis of the state of affairs, production techniques, and waste valorization alternatives. Additionally, numerous KW hydrolysis pre-treatment techniques are discussed. The assessment will help to elaborate on the current state of PHA production from KW, along with addressing any sustainability-related issues.

Microbial bioeconomy can improve through microbial conversion since it has many benefits, such as straightforward fermentation operations, quick cell development, remarkable adaptation to changing environments, controllable product synthesis, etc. Lignin and aromatics mineralize into useful compounds, such as polyhydroxyalkanoates (PHAs), via bacterial strains; several ligninolytic bacteria species have been identified [11].

Under aerobic conditions, these bacteria may break the aromatic rings of lignin-derived aromatics by the -ketoadipate pathway; however, three other methods may be used under low oxygen circumstances. The precursors, including succinyl-CoA and acetyl-CoA, will be produced to create PHAs via the oxidation route or de novo fatty acid synthesis. Additionally, previously published studies have shown that some bacteria release ligninolytic enzymes to speed up the decomposition of lignin [12].

Several bacteria having ligninolytic enzymes, such as high lignin peroxidase (LiP) activity, had been isolated from termite intestines using lignin media. Among these ligninolytic bacteria, *P. putida* KT2440 had the highest ligninolytic capacity adequately examined. The strain screening procedures to discover the ligninolytic strains had not yet been properly developed. Therefore, it is essential to find new strains with strong ligninolytic activity and the engineering potential for industrial application to build an efficient biological lignin valorization. Numerous bacteria produce polyhydroxyalkanoates (PHAs), which serve as an intracellular carbon store and an energy source in the face of hostile environmental conditions. PHAs are straightened polyesters constituted of around 200 fatty acid monomers with different chains of carbon [12].

There are three types of PHAs, including short-chain (scl-) PHAs (C3–C5), medium (mcl-) PHAs (C6–C14), and long-chain-length (lcl-) PHAs (>C15), made up of 3-hydroxyalkanoate monomers. Due to their biodegradability and hydrophobicity, PHAs are currently viable renewable alternatives to petroleum-based polymers. Commercial application of PHAs is difficult due to their poor characteristics and high production costs. A cheap source of lignin utilization would be a significant step to lower the PHA’s production price. The ability of a few lignin-degrading strains to accumulate PHAs and develop an efficient route for lignin metabolism is crucial.

For instance, *P. putida* CA-3 generated a greater yield of medium-chain PHA from styrene in comparison with glucose as a substrate. In addition to producing 0.25 g/L mcl-PHA from the lignin substrates it ingested, *P. putida* KT2440 may make additional PHAs through the combined utilization of sugar and soluble lignin. With monomeric composition, the variety of PHA characteristics increases. Longer carbon chain PHAs are comparably low-density polypropylene due to a low melting point and higher flexible crystallinity. Contrarily, polyhydroxybutyrate (PHB) resembles isotactic polypropylene more closely. PHAs’ monomer structures differ depending on the culture substrates that are employed. *Pseudomonas putida* produced mcl-PHA with a greater mole content of 3-hydroxy decanoate from styrene in comparison with 3-hydroxy hexanoate (a primary monomer of mclPHA) produced from organic acid or glucose.

However, to minimize PHA aggravation in strains and poor PHA homogeneity, the bioconversion of lignin to PHAs is still insufficient. Additionally, the impact of aromatic substrates on the monomer makeup of PHAs has not yet been thoroughly understood. Therefore, it is vital to comprehend how ligninolytic bacteria valorize lignin derivatives and how this affects the variety in PHA composition.

## 2. Present KW Management and Disposal Procedures

Municipal solid waste (MSW) is created at a rate of 2 billion tons annually. According to the World Bank (2018), one person produces about 0.7 kg of garbage every day. In 2018, the Environmental Protection Agency (EPA) reported food waste to account for 21.6% of the 292.4 million tons of MSW produced in the USA. 90% of food waste is generated by the European Union (EU), of which the food sector is responsible for 38%. To deal with food waste, waste management options, including recycling, processing for reuse, separate or mixed collection, and some disposal techniques like landfilling, could be employed [13]. Dumping or disposing of the majority of MSW in landfills is adopted worldwide, accounting for 37% of dumped garbage, with 8% going to sanitary landfills and systems for collecting landfill gas. According to the World Bank (2018), 31% of waste is dumped in open landfills, 11 is burned out, and 19% are recovered using composting techniques and recycling. The advantages of these treatment methods in terms of the environment and economy are mostly dependent on regional variables, such as population, climate, and markets for products (such as energy). There are numerous ways to collect and dispose of KW, but the environmental advantages and cons are still poorly understood. In other nations, the KW is broken up into bits using food waste grinders. A centralized composting system can handle all the organic waste, which could not be achieved by using simple food grinders, although they are easy to use. Similarly, operational and management costs, along with their impact on the system for these grinders, are still debatable [14].

KWs and food residues are found in organic waste released from homes, restaurants, processing facilities, retail locations, and caterers. Japan, Korea, the USA, and India discard about 624–3500 × 104 tons of food waste [15]. The other way of using it is as bioenergy by anaerobic digestion and as fertilizer by composting. Due to their high moisture and salt content, these wastes require a lot of energy to burn, which releases toxic pollution. As a result of several ecological difficulties, governments have implemented severe regulations on KW control and operation since 2002 and outlawed feeding these wastes to animals directly [16]. In 2001, The Law of Promotion and Recycling, along with other related activities to treat cycling food, was published in Japan. This program aimed to decrease the amount of food waste produced and the amount that was turned into fertilizer and livestock feed [17]. The Waste Minimization Plan was put into effect in Malaysia in 2005. It highlighted techniques like composting and aimed for 100% source-to-sink separation of organic waste by 2020 and 20% organic waste recycling [18]. The European Parliament demanded immediate action in 2012 to cut food waste in half by 2050. A goal to cut global food waste per capita in half by 2030 was proposed in 2015 as part of the United Nations (UN) Agenda for Sustainable Development. Food waste was identified by the European Commission (EC) as one of the priority topics of the EU’s Action Plan for Circular Economy in 2015 [18]. The waste management hierarchy developed by the European Union (EU) and the United States Environmental Protection Agency (USEPA) has the concept of reducing, reusing, and recycling food waste. Unfortunately, a major part of food waste is still combined with MSW and dumped without proper, efficient waste management in the majority of nations.

## 3. Ingredients in Kitchen Waste

**Figure 1:**
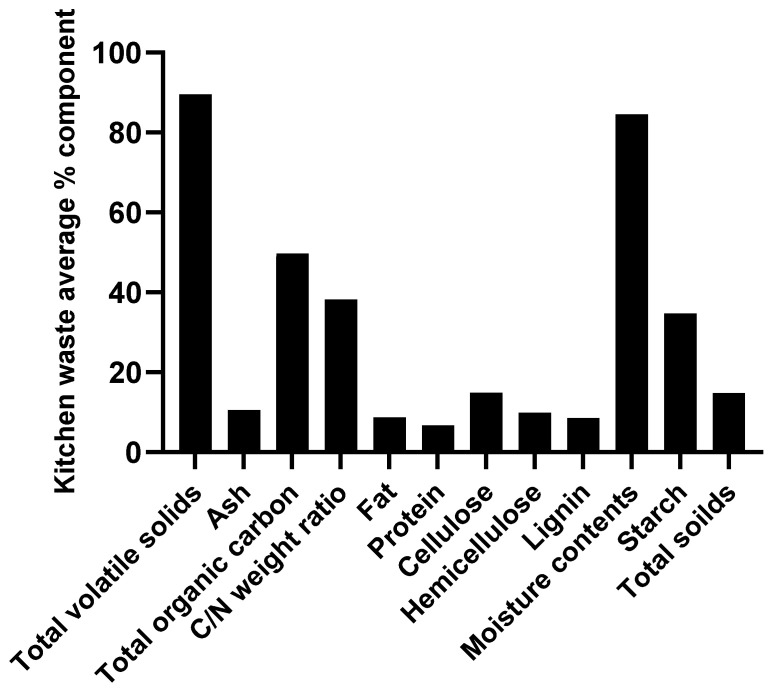
Kitchen waste average percent composition.

The waste produced in the kitchen depends on where it has come from, resulting in variability and the exact nature of the waste being ambiguous to identify across nations. The majority of KW normally consists of rice, meats, and veggies based on published reports. The production stage consists of roughly 2% oil, 4% meat and fish waste, 6% dairy waste, 14% bowls of cereal, 2% roots and tubers, and 48% foods and vegetable waste. Furthermore, Vavouraki reported that different KW’s compositions, including roughly 18.5% solid components, 81.5% moisture, and a pH range between 4 and 6, are regularly generated [19] (Figure 1). These substances have the potential to synthesize high-value products. Nearly 60% of KW’s composition is made up of carbohydrates, 20% of which are proteins, and 10% of which are lipids [20]. Meat and food waste are two of the major parts of KW, which are rich sources of nitrogen and some micro- and macronutrients. Various forms of food waste contain total nitrogen in the range of 1.1% to 9.6%, with an average value of 4.6%. Phosphorus is also found in the ratio of 0.1 to 1.2% in KW [21]. The most important characteristics of KW are its moisture content, humidity, and calorific value. Bacteria can create a variety of biopolymers, including PHA, and biofuels like methane, hydrogen ethanol, etc., upon hydrolysis of KW, which releases simple sugars by fermentation. Therefore, it is crucial to find a technology that directly transforms the organic components of KW into high-value products like PHA to address the issues.

## 4. PHAs and the State of PHA Synthesis

The biodegradability and derivation of the PHAs from renewable resources make them a viable replacement for traditional plastic materials [22]. Certain bacteria can produce these biopolymers in the cytoplasmic granules when they are under stress due to a lack of nutrients [23]. The distinctive properties of these biopolymers, like the stronger oxygen barrier, fat/odor barrier, and water vapor barrier, have led to their employment in a variety of industries, including medicine, energy, fine chemicals, and food packaging. The key determinants of the physiochemical properties in the fermentation process are the bacterial strain and operational parameters applied [24].

The enormous environmental advantages that bioplastics offer are one of the main factors influencing the growing interest in them. Currently, the revenue generated by bioplastics is around USD 5.8 billion greater than that of 2014 [25]. The predicted value of PHA is USD 62 million in 2020, which could be increased by USD 121 million by 2025 [26]. PHA production is now 5–10 times more expensive than that of petrochemical polymers. The anticipated cost of producing raw PHA for both big and small PHA production facilities is 1.3 USD/Kg of unprocessed PHA [27]. As a result, high manufacturing costs decreased further processing effectiveness, and the energy required for the sterilization of fermenters is one of the main obstacles standing in the way of PHA’s market penetration and actual use [28]. PHA manufacturing from home-generated organic waste and agro-industrial waste has become more and more popular in recent years as a means of strategically lowering the costs of production [29].

KW, which is sufficiently rich in nutrients and carbon, could be utilized as the right source for PHA generation by microbial fermentation [30]. To make KW more biodegradable before PHA synthesis, an appropriate pre-treatment procedure is required. In the past few years, most of the scholarly articles and publications focused on the majority of food waste, used palm oil, and used cooking oil. Table 1 shows that used coffee grounds and composite food waste were the wastes most frequently used for PHA buildup. It displays the potential of KW’s features when used to produce PHA.

Other than KW researchers, attention has recently been drawn to the bioconversion of food and kitchen waste by using insects, called insect-based bioconversion. The larva of the black soldier fly is a popular option since it can transform organic waste biomass into high-protein biomass while reducing it by 50–60% [31]. The optimization of insect feed composition, insect selection, insect rearing conditions, and regulatory requirements are necessary for the new approach of insect-based bioconversion, which is outside the purview of this article at the moment.

**Table 1:** Comparison of PHA yields from the studies that used part of KW as a carbon source.

S.No	Substrate	Characteristics of the Substrate	Substrate Pre-treatment	Culture	PHA Production	Extraction/Analysis of the Samples	Fermentation Time (h)	PHA Content (% gPHAs/g Biomass)	Biomass Concentration (g/L)	References
1	Spent coffee grounds (SCG) oil	High amounts of organic fractions like fatty acids and minerals	Supercritical extraction of the SCG oil	Cupriavidus necator DSM 428	After batch processing, there is an N-limited fed-batch step	Precipitation of the polymer after adding n-hexane to the broth	48 h	78.4	16.7	[32,33]
2			Extraction of the SCG oil with the use of n-hexane in an extractor apparatus	Cupriavidus necator H16	Fed batch cultivation	Centrifugation of the samples and washing of the cells with 5% (*v*/*v*) Triton X and distilled water	72 h	90.1	49.4	[33]
3	Used cooking oil	High content of lipid (fat)	nd	C. necator DSM 428	Batch operation with excess nitrogen	Collection of the broth, washing with hexane, lyophilization, extraction with chloroform	50 h	63	11.6	[34]
4	Waste rapeseed oil	High content of lipid (fat)	nd	Pseudomonas sp. G101 and G106	Cultivation of the cells in a nitrogen-limited	Dissolving of PHAs in chloroform. Precipitation with methanol and evaporation	48 h	21	nd	[35]
5	Pure vegetable oil	High content of lipid (fat)	nd	Cupriavidus necator H16	Batch fermentations	nd	24 h	n. r	1.2	[36]
6	Composite food waste	Contains easily consumable volatile fatty acids	Mixing of fresh food waste slurry with the inoculum in a 2 L reactor	Cupriavidus necator	Feeding regimes: pulse stepwise (once a day with 7 pulses) and continuous feeding	Analysis with GC	259 h (8 draw–fill cycles)	87	nd	[37]
7	Restaurant waste	Large proportion of fatty acids, carbohydrate, protein, and fat	Recovering of VFAs by the freezing-thawing method. Centrifugation	*E. Coli* pnDTM2	Batch and feed batch culture in the bioreactor	Determination of PHB concentration by HPLC	At 5th day of fermentation	Batch culture 36.4 Fed batch-44	39.6	[38]
8	Kitchen waste (orange peel and onion peel)	Significant supplies of sugars, lipids, carbs, and mineral acids can be found	nd	Bacterial strains isolated from polluted environments	nd	nd	48 h	82	12.6	[39]
9			Acidogenic fermentation	Cupriavidus necator CCGUG 52238	Batch and feed batch fermentation	Analysis of the PHB content in the cell mass with HPLC	n. r	84.5	230	[40]
10	Waste vegetable oil	High content of lipid (fat)	nd	Isolation of corn oil-degrading bacteria from a rice field	nd	nd	72 h	37.3	0.9	[41]
11	Spent palm oil	High content of lipid (fat)	Addition of spent palm oil to the medium and autoclaving. Addition of sterilized 1,4- butanediol.	Cupriavidus necator	nd	nd	n. r	81	12.5	[42]
12	Food waste compost (FWC)	Significant supplies of sugars, lipids, carbs, and mineral acids can be found	Acidogenic fermentation in an anaerobic bioreactor.	Mixed microbial culture	Acidogenic fermentation in anaerobic bioreactor	nd	12 h	23.7	nd	[43]
13	Food waste compost (FWC) Unfermented		Mastication, filtration, oil removal, and dilution in domestic sewage to the required OLR	Mixed microbial culture (aerobic mixed culture)	SBR1: unfermented food waste/aerobic microenvironment	nd	48 h	35.2	nd	[44]

nd: not determined.

## 5. Available Carbon or KW’s Capacity for Biodegradation

*Cupriavidus necator* produced the greatest concentration of 4.1 g/L of PHA in an investigation into the economic feasibility of generating PHA utilizing KW as a substrate [45]. In the synthesis of commercial PHA, the majority of the carbon used comes from fatty acids and mono- and disaccharide sugars. However, the starch, lignin, and cellulose components found in KW constitute the carbon source, and these substances are difficult for bacteria to convert into PHA [46]. Therefore, kitchen waste containing these carbohydrate polymers must be hydrolyzed into fermentable sugars to produce bacterial PHA [47].

It is advised to utilize pre-treatment methods such as physical, chemical, or enzymatic reactions to depolymerize the polymers before they reach the hydrolysis stage, such as cellulose, lignin, and hemicellulose [48]. Amylase enzymes, with two distinct categories, amylase (EC 3.2.1.1) and glucoamylase (EC 3.2.1.3), are being researched to be utilized for the hydrolysis of kitchen waste. By cleaving 1,4-glucosidic links, carbohydrates are converted into glucose, maltose, and maltotriose by the hydrolytic activity of amylase, whereas both 1,6- and 1,4-glucosidic connections are cleaved at the branching site of amylopectin, forming glucose as the end product [49]. Waste produced by fruits and vegetables can be successfully used as biomass feedstocks in the microbial fermentation process to create PHA. These wastes have a high sugar content (between 4 and 12.8 g/100 g) but a poor protein content (between 0.8 and 2.6 g/100 g) [50].

The *Cuprividus necator* and strain SPY-1 showed PHA content of 41.7 and 42.2% *w*/*w* upon hydrolysis when Jambul seeds were used in fermenter media as carbon feedstock [51]. Apricots, cherries, and grapes were among the nine various types of pomace fruit wastes that was utilized as carbon sources [52].

In addition, 10.2 g/L was the ultimate biomass concentration, and the final mclPHA content was 12.4% *w*/*w* or 0.03 g/Lh-1 of mclPHA volumetric production output. The investigators also found that all the fruits, including apricots, had the fewest development inhibitors. Cruz used coffee grounds from kitchen waste as a unique carbon source when cultivating *Cupriavidus necator* DSM 428. 0.2 g/Lh productivity was generated from culture and a polymer content of 78.4% [32]. Animal products, such as leftover meat and fish, can also be used as feedstock for the manufacture of PHA. By using animal manure, scientists were able to generate bioplastic concentrations of 0.04 and 0.05 g/Lh1 with PHA levels of 20.1% and 26.6%, respectively [53]. A comparison of the results from experiments using kitchen garbage as a carbon source is shown in Table 1.

In addition, dairy products, some vegetables, some fruits, derivatives of meat, and some dairy products are key sources of nitrogen, phosphorus, and minerals in KW, which are essential ingredients for PHA production. Since KW may be used to generate PHA, it can be said to be an acceptable source of nitrogen, phosphorus, and other micro- and macroelements. The buildup of PHA and unsaturated fatty acids depends on the carbon-nitrogen (C/N) ratio and, in some cases, the carbon-to-phosphorus (C/P) ratio [54]. At a C/N ratio of 100, the highest PHA accumulation was attained. The same results were reported, who discovered a biomass PHA content of 74.6% and a C/N/P ratio of 100:3:1 [55]. Thus, it can be inferred that N constraints encourage PHA and fatty acid synthesis in oleaginous bacteria. To investigate the effect of the C/N ratio in the hydrolysates of rice straw on the PHA accumulated by CN, by utilizing glucose and NH2CL [56]. There was a higher rate of PHA synthesis when exposed to N-deficient conditions (such as a C/N ratio of 3.6:1 or 36:1) as compared to exposure to extreme N-poor situations (e.g., a C/N ratio of 360:1). However, it can be difficult to use complicated substrates, such as KW, because the N content needs to be controlled.

To improve the C/N ratio from 20 to 35, glycerol was added to the yeast lysate in one study, which multiplied the yield of the finished product by seven [57]. Additionally, soy waste is also used as a carbon source to synthesize PHA at different C/N ratios [58,59]. AA polymer with a high hydroxybutyrate (HB) mole fraction (hydroxybutyrate:hydroxyvalerate, 75:25) was created using soy waste as a carbon source. KW is also a viable source of P and metal profiles that include sodium, potassium, calcium, and magnesium as significant components, along with trace elements like zinc, manganese, and iron and heavy metals like copper, nickel, and arsenic, etc. For bacterial growth and PHA synthesis, several of these ions and trace metals may be crucial.

## 6. KW Hydrolysis

The degree of carbohydrate saccharification affects how effectively KW is converted to PHA [46]. Furthermore, the complex and heterogeneous nature of KW results in low hydrolysis of the sugars produced. Furthermore, the biochemical methods do not show the influence of each component of kitchen waste on the pace and degree of hydrolysis. The degradability of carbs, proteins, lipids, and fiber also varies due to physicochemical and structural variations, which will be covered in more detail in the paragraphs that follow.

The end product of carbohydrate hydrolysis in an acidic environment is a heterogeneous blend of simple sugars and disaccharides. Amylase enzymes were of focus in already existing studies for the saccharification of carbohydrates due to their critical functions in limiting the conversion of large molecular masses of complex sugars [60]. Additionally, KW has a sizable amount (6.7%) of proteins because of the presence of meats, fish, eggs, beans, and chicks. When compared to the breakdown of sugar and fat, protein disintegration is said to be a sluggish process [61]. Animal excrement can be hydrolyzed chemically into amino acids in both acidic as well as in alkaline conditions, or by enzymes like pancreatin and pepsin, etc.

Several volatile fatty acids (VFAs), which are precursors to the synthesis of PHA, are produced by the hydrolysis of proteins. These VFAs include branched-chain fatty acids, lactic acids, propionic acids, acetic acids, butyric acids, and a small quantity of methane, carbon dioxide, hydrogen, ammonia, and sulfur [62].

A total of 12–15% (*w*/*w*) KW is made up of crude fat together with proteins [63]. Increasing temperature speeds up the endothermic reaction of fat breakdown, sometimes referred to as lipolysis. VFAs, which are the starting point for the synthesis of PHA and glycerol, are the main byproducts of fat hydrolysis [64]. At high temperatures, the fats and oils undergo thermal disintegration, ultimately resulting in lower free fatty acid yields. Fatty acids are the rate-limiting stages in the breakdown process of KW since we know the breakdown of crude fat is slower than carbohydrate degradation [65]. Another issue of bioreactor clogging, mass transfer, and flotation is caused by the presence of fats in KW, which lowers the efficiency of the hydrolysis process [66].

**Figure 2:**
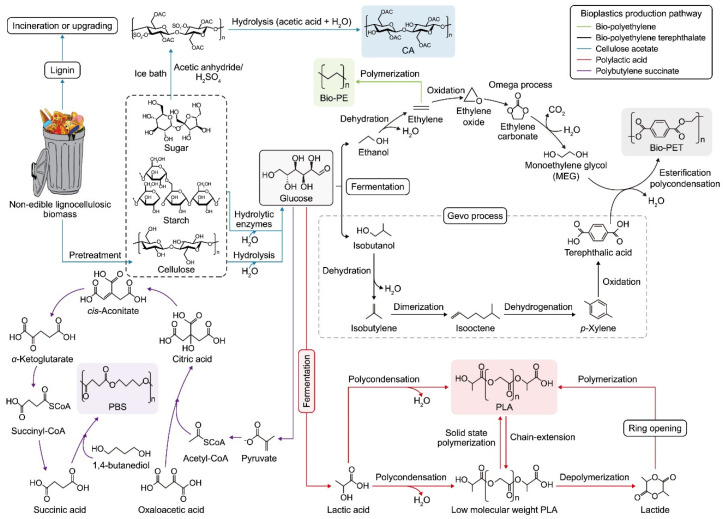
Polylactic acid (PLA), polybutylene succinate (PBS), cellulose acetate (CA), bio-based polyethylene terephthalate (Bio-PET), and bio-polyethylene (Bio-PE) production pathways from non-edible lignocellulosic biomass.

In addition to simple sugars, KW also contains insoluble polysaccharides, such as cellulose and hemicellulose, which comprise the fibers primarily present in the peels of fruit pieces and vegetables (Figure 2). Cellulose fibers are made up of parallel, unbranched Dglucopyranose units that are joined by 1,4-glycosidic linkages to produce crystalline, highly structured microfibrils [67]. They can also work as a source of carbon for microbial PHA buildup since they are hydrolyzed into simple sugars [68]. Glucose could be recovered from hydrolysate in the range of nearly 40–60% after pre-treating KW with acid [69].

High ionic salts like sodium are also found in kitchen waste, which is frequently used in Asian countries as a flavor enhancer. Because of the complex security concerns, the presence of salt prevented the use of pre-treated KW as animal nutrition [70]. Microbial development in the culture is substantially affected by increased osmotic pressure due to salts like Fe^3+^ and AL^3+^. Furthermore, PHA production is inhibited by high Na+ ion concentrations. Therefore, to achieve high PHA output, the treated KW must be examined carefully for ionic content.

## 7. Various Pre-Treatment Techniques to Enhance Nutrients Available for PHA Production

1. Mechanical and/or heat procedures carry out the physical pre-treatment method. To achieve high biological conversion, speed up the separation, and increase surface area for improved degradability, biomass can be processed by using microwaves, ultrasound, chipping, milling, grinding, heating, etc. Physical pre-treatment techniques are typically employed in conjunction with other techniques to reduce particle size or separate the materials at the beginning of the process [71]. Izumi and their team, looked at the impact of solubilizing and reducing the particle size of food waste [72]. Upon reducing the waste size from 0.8 mm to 0.4 mm, the amount of organic stuff that could be dissolved rose by 30%. Radiation-based physical pre-treatment cleaves 1,4-glycosidic bonds, increasing surface area and decreasing crystallinity. However, it is an expensive approach with significant safety and environmental issues. Solid waste is pre-treated by hydrothermal treatment to increase its digestibility. Kitchen trash has been subjected to hydrothermal pre-treatment at various temperatures, including 37 and 60 °C, to determine the degree of hydrolysis. Findings show that 60 °C is the most efficient temperature for hydrolyzing about 27.3% of kitchen waste and can remove 37.7% of oil and grease [73]. In another investigation, pre-treatment at 160 °C for 5–50 min was done using a hydrothermal pre-treatment strategy, increasing the glucose concentration from 37.5% to 43.9% after that time [74].

2. For lignocellulosic substrates, chemical pre-treatment is most typically utilized to improve biodegradability. Acid hydrolysis is frequently employed because it can be used with a variety of feedstocks. Concentrated acid treatment is not preferred over dilute acid treatment for industrial applications because dilute acid treatment requires less expensive maintenance and operational processes, produces fewer inhibitors, and can recover up to 80% of the hemicellulose sugars. Pre-treatment options like chemical and/or thermal are usually recommended to synthesize volatile fatty acids from food wastes generated [75]. Another study looked into the physicochemical pre-treatment of KW using various chemicals, such as H_2_SO_4_, HCl, NaOH, and H_2_SO_3_, at varying temperatures (50, 75, and 120 °C) and incubation times (30–120 min). The findings employing either 1.1% HCl (94 min) or 1.2% HCl (86 min) at 100 °C indicated an increase in soluble sugars by 120% as compared to untreated KW [19]. Alkali and alkaline treatment options were not so effective.

3. Enzymatic pre-treatment techniques, which utilize enzymes from microbes such as peptidases, sugars, and lipases, are promising environmentally friendly techniques. The hydrolytic ability of KW is improved by enzymatic pre-treatment, which also reduces volatile suspended particles [76]. Hydrolysis of 1,6-glycosidic linkages in polysaccharides can be catalyzed using amylase to produce linear oligosaccharides in KW with high starch content. Glucoamylase can produce the fermentable sugars glucose, sucrose, fructose, and maltose. Cellulolytic enzymes, such as endoglucanase, exoglucanase, and glucosidase, are necessary in cellulose-rich fractions [15].

Volatile suspended particles, compared to glucose and lipase, were lowered by protease. Better efficiency, lower energy needs, lower corrosion impact, and less toxic compound generation are some benefits of enzymatic pre-treatment over chemical pre-treatment [77]. Enzymatic therapy is less desirable on an industrial scale due to slow reaction rates and tight condition control, though. Additionally, expensive enzymatic pre-treatment needs high enzyme concentrations to be effective [78]. However, it has been investigated that employing in situ-produced waste enzymes could lower the cost of enzymatic pre-treatment [79].

## 8. The Effect of KW Pre-Treatment on the Formation of Byproducts and Inhibitors

Hexose and pentose are the primary types of sugars released when carbs and dense biomass are digested [80]. 5-hydroxymethylfurfural (HMF), furfural, and volatile fatty acids (VFA) can also be created by excessive hydrolysis brought on by harsh treatment conditions, which lowers pH and, as a result, prevents KW from hydrolyzing.

The majority of these chemicals are created during the lignocellulosic materials’ thermal and acidic pre-treatment [81]. The Millard reaction occurs when sugar reacts with protein complexes at high temperatures, resulting in the accumulation of melanoidins or amadori substances, which are seen in the brown-colored hydrolysate compounds [82]. It is believed that the Maillard reaction is the main reason why carbohydrates are lost during hydrolysis. The breakdown of hexose produces major byproducts, including VFAs and furans like HMF, and when HMF is transformed in an acidic environment, levulinic acid and formic acid are produced in a 1:1 mol ratio [83]. Similarly, formic acid and other byproducts are produced by the breakdown of pentoses like xylose and arabinose into furfural [80]. Figure 2 depicts the intricate molecular process of kitchen waste hydrolysis [84].

The concentration of the byproducts depends significantly on variables like temperature, time, biomass type, and solid concentration. El-Tayeb, looked at the development of HMF during the hydrolysis of several types of food waste with H2SO4 (15% *v*/*v*) at 120 min [85]. They found that the buildup of HMF increases as the number of acids increases. Furfural and HMF are frequent inhibitors that reduce PHA output, particularly when used with the bacteria Escherichia coli, Rhodococcus, and *Cupriavidus necator* [86].

Based on Hafid’s finding, furfural affects the tricarboxylic acid (TCA) cycle and glycolysis enzymes [15]. Additionally, it results in the production of ROS, which harms yeast cell membranes such as those in the mitochondria and vacuoles [87]. According to the most recent research, cellular physiological functions are degraded by HMF and furfural, preventing the growth of microbes by impeding messenger ribonucleoprotein (RNP) assembly and interfering with the process of fermentation for the synthesis of various products [88]. The breakdown of lignin in kitchen waste results in several phenolic compounds. They mostly come in three different shapes: ketones, aldehydes, and acids [89]. Even in low quantities, phenolic chemicals are far more dangerous than other inhibitory drugs.

The scientific team decreases volumetric output by slowing down microbial development and extending the lag period. They infiltrate microbial cell membranes, disrupting them and altering the architecture and activities of microbial cells [90]. This limits microbial growth as well as sugar assimilation and can even cause DNA disintegration, which prevents the synthesis of RNA and proteins. They frequently severely inhibit cellulolytic enzymes as well. Phenolics with low molecular weight can access the enzyme’s active site more easily than phenolics of high weight. Additionally, researchers found that the interaction of phenolics with high carbonyl content and hydrophobicity is strong with cellulase enzymes as well as amino acids [91].

## 9. Recent Studies on Using Leftover Food from the Kitchen as a Source of PHA

Kitchen waste was rarely used for the production of PHA in the literature. The majority of research includes waste from the kitchen and food, such as leftover fruit, leftover vegetables, leftover oils, etc. This section highlights the potential of kitchen waste as a substrate for the synthesis of bioplastics discussed in previous research. Waste cooking oil (WCO) is the oil left after frying food at high temperatures. WCO contains several harmful components, such as aldehydes, ketones, alkanes, hydroperoxides, and fatty acid oligomers, and as a result, they cannot be used for further human consumption. Global production of WCO ranges from 41 to 67 million tons per year [92]. The US and Europe produce 11 billion liters and 1 billion liters of WCO each year (reported by the Energy Information Administration, EIA). One of the issues is how to safely dispose of WCO because their presence in the environment leads to pollution, water deoxygenation, and toxicity due to high BOD and COD. Currently, the manufacturing of biodiesel uses 90% of the WCO that has been gathered [93]. One of the potential uses of WCO is for the microbial fermentation of value-added products. Several studies have demonstrated the potential of employing WCO directly as a raw material for PHA manufacture [94].

In a study, *Cupriavidus necator* was used to create poly(3-hydroxybutyrate-co-4-hydroxybutyrate) [P(3HB-co4HB)] with a cell dry weight (CDW) of 5 g/L and a PHA content of 81% (*w*/*w*) following a 144-h fermentation with WCO and 1,4-butanediol. In comparison to glucose, which yielded 0.3–0.5 g/g of PHA, WCO produced 0.7–0.8 g/g of PHA [95,96]. *Cupriavidus necator* grown on WCO as the only carbon source had a high CDW of 25.4 g/L and a biomass PHA content of 71% [97]. *Cupriavidus necator* H16 generated 80% *w*/*w* content of PHA with 105 g/L concentration when grown using oil propanol repressed on waste. When grown on frying oils, other microbial strains, like *Pseudomonas aeruginosa*, displayed a 53.2% PHA concentration [98]. When valerate was fed with WCO and PHA content of more than 50.1 mol%, HV was produced by *Halomanas* hydrothermally [99]. The screening of *Halomonas* strains to determine their potential to make PHA.

Wastes from the food industry have been identified in numerous studies as potential PHA feedstock. Fruit wastes, among them, have demonstrated excellent potential as a cheap source of carbon for the synthesis of PHA. For instance, the production of wine results in significant amounts of solid waste, primarily made up of grape pomace. It can function as an excellent fermentation substrate because of the high amounts of glucose and fructose. Depending on the grape variety and the date of harvest, the sugar content of grapes (peels and/or whole grapes) ranges from 14.2 g to 67.5 g of glucose per kilogram of grapes [100]. Grape peels contain a significant quantity of lignin (30% of the dry weight), so a procedure (detailed in earlier sections) is required. Using *Cupriavidus necator*, PHB generate using grape extract [101]. A high PHB content was achieved at about 63% in 29.5 h.

The production of large quantities of apple pomace, a waste product made up of the seeds, stem, pulp, peels, and core of apples, has also been reported in the apple juice industry. Fruit waste is typically thrown away or used as animal feed, not for other highly profitable uses. It has large amounts of sugars that bacteria can use either immediately or after pre-treating [11]. To cultivate *Pseudomonas chlororaphis* and manufacture mcl-PHA, discarded apple pulp produced during the production of juice was employed as a source of glucose and fructose [102]. Three-hydroxy decanoate, three-hydroxy octanoate, three-hydroxybutyrate, three-hydroxy tetra decanoate, three-hydroxydodecanoate, and three-hydroxyhexanoate made up the PHA that was created. The annual global production of citrus fruits such as oranges, mandarin lemons, etc. is over 120 million tons. The typical dry mass composition of the residues after citrus fruit juice processing is as follows: 19% pectin, 30% sugars, 12% hemicellulose, and cellulose. The remaining components are oil, proteins, ash, and organic acids. For the first time, investigated the activity of *Bacillus* sp., which can cultivate and utilize the hydrolyzed citrus pulp as the feedstock of carbon to form a PHA of 47.5% of CDW [103]. Simple sugars, including fructose, sucrose, and glucose, compose 2.1% *w*/*w* in banana pulp and 0.45 *w*/*w* in peel, which is thought to be very less than other considered fruits.

*Cupriavidus necator* has been employed by researchers to make PHA utilizing extracts from banana fronds as a carbon source [104]. The greatest PHB content in biomass of 32.1% and 0.9 g/L was achieved when fermentation was carried out using 40% *v/v* extract. Similarly, the PHB content rose to 37.4% and the PHB concentration to 1.3 g/L when banana extract was processed. Fruit type, the fruit portion to be treated, and the harvesting season all affect how feasible it is to use fruit wastes as a substrate in the fermentation process. Furthermore, the determination of other factors like sugar content, composition of monomers, and nitrogen content is also needed, as they can influence the PHA yield and its monomers. Hidayat utilized the hydrolysate of oil palm empty fruit bunch as a carbon substrate for the synthesis of PHA by *Bacillus cereus* B-001 [105]. To obtain fermentable sugars, the fruit waste was first processed with H_2_SO_4_, and then an alkaline solution was used for neutralization. PHB generation was 55.4% of CDW when these hydrolysates were employed, as opposed to 43.1% with glucose. Using fruit waste, researchers have also investigated the halophile *Haloferax mediterranei* for PHA buildup [106]. Poly(3-hydroxybutyrate-co-3-hydroxyvalerate) was created by a bacterium using date fruit garbage as a source of carbon and other components. About 18 g/L CDW with a 25% *w*/*w* concentration of PHA was attained in fed-batch fermentation.

## 10. Combined Food Waste and Leftover Meat or Seafood

Due to the growing concern about the development of food waste worldwide, using bio-composite trash could be a profitable and practical solution to create high-value products. Based on Choudhary research, fruit peels such as pineapple, jackfruit, banana, apple, and sugar beet, and seeds like mango, papaya, etc., along with egg and fish shells, comprise the majority of the food waste composite [107]. Composite waste is to be hydrolyzed to create volatile fatty acids. To reach the requisite organic load rate (OLR), other investigations included oil removal, sample preparation, and pH adjustment in sewage sludge [43], whereas composite food waste was neutralized in a study by a team [38]. To learn more about how temperature and pH affect bio-fermentation, different temperature and pH regimes were used during the fermentation. The acidogenic fermentation has occasionally been carried out by anaerobic consortia [44]. They also produced PHA using recombinant E. coli from fermented restaurant composite waste, and they found that the PHB level was 44% (*w*/*w*). Scientists used the same substrate [39], but they found a lower PHA content than that of the reference strain [108]. They also showed that a bacterial isolate employing onion peels as a substrate produced more PHA.

Another significant class of complex nitrogen sources is waste from the meat and fish industries. The main advantage of using various nitrogen sources is its potential to reduce the large phase at the start of the fermentation process. This is caused by the availability of a complex nitrogen supply that can be readily transformed by bacterial cells into proteinaceous material. As a result, more catalytically active biomass accumulates PHA in a very short time. In another investigation, solid waste produced by fish was used as substrate, and that of *Bacillus subtiltis* was used to create PHB. In addition, 1.62 g/L of PHB was produced [109]. In comparison to control cultivation, these substrates increased PHA generation by 50% [110].

The aforementioned investigations show that KW can produce bioplastics and that it can be used to lower process costs. Synthesis of PHA from whole kitchen waste on direct usage; however, no research has been documented. Therefore, research should be directed on this side to make the process stronger and more sustainable.

## 11. Technical Challenges and Possible Results for Long-Term KW

**Figure 3:**
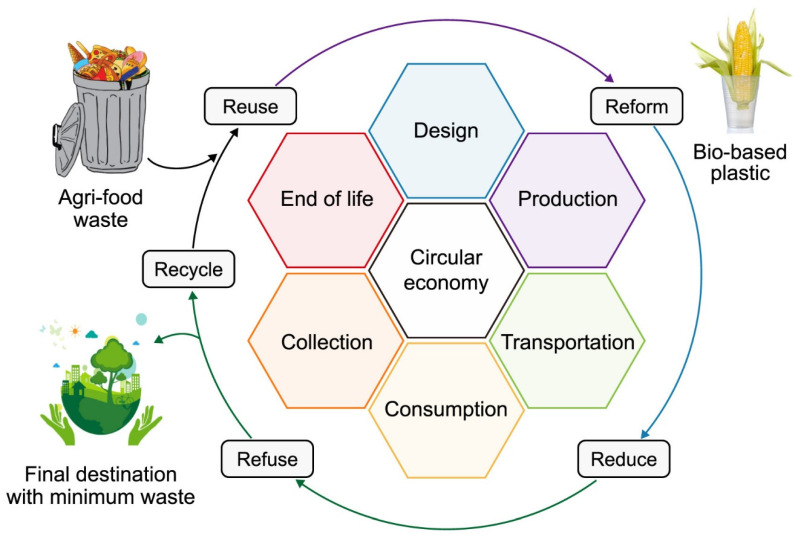
Circular plastic economy concept and bioplastic End-of-life.

The idea to turn solid waste into useful and environmentally friendly products has paved the way to safeguard the environment and ecosystems and provide green solutions (Figure 3). It aspires to remodel waste management systems while reducing negative potential through reimagining products and services via system-wide innovation. The risk of a food-fuel conflict may be reduced by using KW (whole or in part) for the production of PHA, as outlined in the previous sections. The unlimited resources that are evenly dispersed globally for fossil fuels are the main benefits of employing KW for biopolymer manufacturing [111]. However, there are a few issues with KW use over the long term, which are outlined below. Due to its composition and complexity, especially when compared to other typical biomasses like crops and lignocellulosic materials, KW is an unconventional feedstock. Additionally, the variety in the locations where it was produced and the locals’ culinary preferences account for the diversity of KW components. To enable a more precise selection of the pre-treatment approach, a suitable characterization method must be provided. To separate KW’s constituent parts, like C and N, for high-value compounds, there may be considerable processing expenses involved. To develop a concurrent technique to synthesize PHA from kitchen waste, a single bioreactor could be employed, which involves encouraging the use of the full KW waste. Due to its composition and complexity, especially when compared to other typical crops like lignocellulosic materials, KW is an unconventional feedstock [112].

Household biodegradable trash needs to be properly distinguished from other recyclable materials to promote a waste-to-wealth strategy. The described KW can be sent to various recycling facilities, where they will be used as feedstock for the manufacture of biopolymers [113].

It is noted that before mixing the kitchen waste with municipal garbage, it is not stored correctly. Facilities to separate and collect the garbage must be installed, notably for KW to augment the current waste management system, due to public behavior. Local governments dealing with the Department of Housing and municipal authorities should play significant roles in constructing a successful plant and collecting system [114]. To improve municipal sales recovery, in countries like Malaysia and Thailand, their governments have introduced trash separation using different containers to separate organic and recyclable wastes [115]. A corporate collaboration of many experts from several disciplines, such as climate engineering, downstream processes, biological and chemical catalysts, and biotechnologists, is necessary to continue utilizing an inclusive approach to the production of bioplastics. Microbial stability is another crucial component of KW that needs to be addressed; in KW, both fungus and bacteria display traits of microbial communities. KW typically has low pH values, which are also associated with higher concentrations of lactic acid bacteria. The metabolic changes that occur during the various stages of KW storage and its microbial degradation process are included. Therefore, a thorough microbiological examination of KW is necessary because these organisms can act as indicators of KW composting and, eventually, of efficient bio-energy production.

Even though KW is affordable, easily accessible, and inevitable, the main costs involved with using KW are its classification, shipping, and pre-treatments. Due to the lack of thorough publications in this area, a techno-economic analysis must be done to establish whether the technology is commercially viable. In this assessment, in-depth information on the process of flow, profitability reporting of the manufacturing plant, actual market statistics, layout, and expense estimate of the PHA facility is reported. An effective and profitable pre-treatment and hydrolysis process is needed for the conversion of biomass to sugar [116]. Enzymes are often less competitive in large-scale manufacturing because of their higher prices, lower sugar yields, and slow reaction times. Pilot-scale production of fermentable sugar is crucial to improving the commercial-scale generation of bioplastics [117,118,119,120,121,122,123,124,125,126,127,128,129,130].

## 12. Conclusions

The microbes utilized to transform waste food into PHAs are distinctive, ranging from identified, well-defined microorganisms to complex and mixed microbial consortia. As specified, both indigenous and genetically engineered PHA strains could be used as platforms for PHA production. Strain choice and selection are critical elements of PHA that could provoke more useful microbes in the future for enhanced PHA production. An innovative and long-term strategic strategy to create a clean, sustainable, and long-term energy source may involve utilizing KW as a substrate for PHA manufacturing. This approach also works well in reducing the existing dependency on fossil fuels and can help with several waste management problems. However, more study into PHA synthesis utilizing microbial methods is required because KW’s form, range, and composition vary in nature. However, using KW in the microbial fermentation process to create bioplastics will be a crucial step toward the application of green fuel and polymers in a sustainable way.
